# Metabolic reprogramming in cancer-associated fibroblasts: orchestrating the tumor immune microenvironment and response to immune checkpoint blockade

**DOI:** 10.1186/s40164-026-00837-6

**Published:** 2026-10-10

**Authors:** Luoman Gan, Zhonglong Liu, Liren Cao, Xiaoyan Meng, Xuanhao Liu, Xu Wang, Yue He

**Affiliations:** Department of oral maxillofacial & head neck oncology, Shanghai Ninth People’s Hospital, Shanghai Jiao Tong University School of Medicine; College of Stomatology, Shanghai Jiao Tong University; National Center for Stomatology; National Clinical Research Center for Oral Diseases; Shanghai Key Laboratory of Stomatology, No.639 ZhiZaoJu Road, Shanghai, 200032 P. R. China

**Keywords:** Cancer-associated fibroblasts, Metabolic reprogramming, Tumor microenvironment, Immunotherapy, Immune resistance

## Abstract

Immune checkpoint blockade (ICB) has reshaped the therapeutic landscape of solid tumors. However, durable clinical benefit remains limited to a subset of patients because of primary or acquired resistance. Beyond tumor-intrinsic factors, the tumor microenvironment (TME) plays a critical role in shaping responsiveness to immunotherapy. As a major stromal component, cancer-associated fibroblasts (CAFs) contribute to immune suppression through dynamic metabolic reprogramming and modulate the spatial organization and functional state of immune cells. Recent advances in single-cell transcriptomics, spatial omics, and metabolomic profiling have provided new insights into the metabolic heterogeneity of CAF subsets and their interactions with immune cells. Metabolic reprogramming not only sustains CAF activation but also remodels the local immune environment through nutrient competition, metabolite accumulation, and coordination with extracellular matrix remodeling. These processes reinforce immunosuppression within the TME and contribute to resistance to ICB. This review integrates emerging multi-omics insights into CAF metabolic heterogeneity, summarizes how metabolic reprogramming shapes tumor immunity, and discusses therapeutic strategies targeting CAF metabolism to enhance immunotherapy efficacy, together with the major challenges facing clinical translation.

## Introduction

Cancer immunotherapy has become an integral component of modern cancer treatment. Among currently available immunotherapeutic strategies, immune checkpoint blockade (ICB) has been widely adopted for the treatment of solid tumors and has significantly improved long-term survival in a subset of patients [1–3]. Nevertheless, therapeutic responses remain highly heterogeneous, with both primary and acquired resistance posing major clinical challenges [4, 5]. Increasing evidence suggests that this heterogeneity in treatment response cannot be explained solely by tumor-intrinsic genetic alterations. Instead, the tumor microenvironment (TME) plays a pivotal role in shaping immune cell composition, functional states, and spatial organization [6, 7], thereby influencing clinical responses to immunotherapy [8]. This immune landscape is shaped not only by immune cells but also by diverse stromal populations, including fibroblasts and mesenchymal stromal cells [9–11].

Among these stromal populations, cancer-associated fibroblasts (CAFs) represent one of the most abundant and phenotypically diverse cell populations across solid malignancies [12]. Once regarded as passive structural elements, CAFs are now recognized as key regulators of the TME. Through extracellular matrix (ECM) remodeling [13, 14] and extensive crosstalk with tumor and immune cells [15], CAFs shape the immune landscape and contribute to the establishment of distinct immune phenotypes. Importantly, CAFs do not display uniform pro- or anti-tumorigenic effects [16]. Instead, CAF functions are highly heterogeneous and context-dependent, suggesting that their influence on the immune microenvironment is dynamic rather than static [17].

A key mechanism underlying CAF heterogeneity is metabolic reprogramming. Unlike quiescent fibroblasts in normal tissues, CAFs undergo profound metabolic adaptations in response to tumor-derived signals, hypoxia, inflammatory cues, and other stress conditions [18–20]. Beyond supporting their own survival and secretory functions, these adaptations enable CAFs to actively remodel the metabolic landscape of the TME, with profound implications for antitumor immunity [21]. Distinct metabolic states can differentially influence immune cell recruitment, activation, differentiation, and spatial organization within the TME [22, 23]. Although certain CAF subsets may support antitumor immunity under specific conditions [24, 25], current evidence predominantly implicates metabolically reprogrammed CAFs in the establishment of immunosuppressive microenvironments and resistance to immunotherapy [26]. Consequently, understanding how metabolic heterogeneity shapes CAF-immune interactions may provide new opportunities to improve responses to immunotherapy.

In this review, we summarize current advances in CAF heterogeneity and plasticity, with discussion of the mechanisms driving metabolic reprogramming of CAFs. We then explore how metabolically distinct CAF states interact with immune cells to shape the tumor immune microenvironment and influence responses to immunotherapy. Finally, we highlight emerging therapeutic strategies targeting CAF metabolism and consider their translational potential for improving cancer immunotherapy.

## Heterogeneity and plasticity of CAFs

### Origins and heterogeneity of CAFs

CAFs broadly encompass all fibroblasts activated within the TME and represent a highly heterogeneous stromal population arising from diverse cellular origins [27, 28]. Among these, tissue-resident fibroblasts (TRFs) are considered the predominant source of CAFs in many solid tumors [29]. In pancreatic ductal adenocarcinoma (PDAC), tissue-resident pancreatic stellate cells (PSCs) represent a major source of CAFs and exhibit substantial phenotypic plasticity in response to local microenvironmental signals [30, 31]. In addition to resident fibroblasts, epithelial cells may acquire CAF-like characteristics through epithelial-to-mesenchymal transition (EMT) [32, 33], while endothelial cells can contribute to CAF formation through endothelial-to-mesenchymal transition (EndMT) [34, 35]. Besides, bone marrow-derived mesenchymal stem cells (MSCs) can be recruited to tumor sites and differentiate into CAF-like cells [36]. Consistently, a lineage-tracing analysis in an inflammation-associated gastric cancer model revealed that a considerable proportion of CAFs originated from bone marrow-derived MSCs [37]. Pericytes have also been identified as a potential source of CAFs. Genetic lineage-tracing in NG2-CreERT2 mice revealed that tumor-associated pericytes can undergo pericyte-to-fibroblast transition and acquire fibroblast-like and myofibroblastic features within the tumor stroma [38].

However, lineage diversity alone is insufficient to explain the heterogeneity of CAF populations. Some of this diversity may predate tumor development and reflect the intrinsic heterogeneity of fibroblasts within the tissue of origin. In the mammary gland, distinct CD26^+^ and CD26^−^ fibroblast populations preferentially give rise to different CAF subsets during tumor progression, providing direct evidence that pre-existing fibroblast programs can shape subsequent CAF states [39]. Similarly, lineage-tracing studies in pancreatic cancer have shown that Gli1^+^ and Hoxb6^+^ fibroblasts represent distinct populations in the healthy pancreas, with Gli1^+^ fibroblasts preferentially expanding and contributing to the tumor stroma during carcinogenesis [40]. These findings suggest that the composition and intrinsic properties of resident fibroblast populations can influence the emergence and organization of CAF states.

CAF heterogeneity is also reflected in the spatial organization and functional specialization of fibroblast subsets within individual tumors. In human lung tumors, integrated single-cell and spatial analyses identified two CAF populations co-expressing α-smooth muscle actin (α-SMA) with either myosin heavy chain 11 (MYH11) or fibroblast activation protein (FAP), both of which were associated with T-cell exclusion. MYH11⁺α-SMA⁺ CAFs were more prevalent in early-stage tumors and formed a single-cell layer around tumor aggregates, whereas FAP⁺α-SMA⁺ CAFs were enriched in advanced tumors and formed stromal patches or multiple layers around tumor nests. These populations also displayed distinct matrix programs: MYH11⁺α-SMA⁺ CAFs preferentially produced type IV collagen, whereas FAP⁺α-SMA⁺ CAFs preferentially produced types XI and XII collagen [41]. An integrative analysis of single-cell spatial multi-omics datasets across cancer types further identified four conserved CAF subtypes occupying distinct niches: tumor-adjacent s1-CAFs with contractile and ECM-remodeling programs, interstitial s2-CAFs with matrix- and cytokine-associated programs, perivascular and myeloid-associated s3-CAFs, and antigen-presentation-associated s4-CAFs colocalized with lymphoid aggregates and tertiary lymphoid structures [42]. These findings demonstrate that CAF heterogeneity extends beyond cellular origin and transcriptional identity to encompass spatial positioning, ECM organization, cellular neighborhoods, and immune-regulatory functions.

Beyond fibroblast-intrinsic diversity within individual tissues, CAF abundance and composition also differ substantially across tumor types [43, 44]. Several mechanisms may account for these intertumoral differences. First, CAF accumulation is influenced by the tissue context in which tumors arise. Tumors developing in fibrosis-prone or wound-repair-active tissue may more readily promote fibroblast activation and expansion during malignant progression [45, 46]. PDAC exemplifies this pattern, with abundant activated fibroblasts and ECM deposition forming a highly desmoplastic stroma [47, 48]. Second, stromal-activating signals vary across tumor types. In pancreatic cancer, epithelial-derived Hedgehog ligands primarily activate signaling in the stromal compartment [49], whereas in lung adenocarcinoma, zinc finger E-box-binding homeobox 1 (ZEB1)-high cancer cells induce a secretory program that reprograms CAFs and directs their localization to the tips of invasive projections [50]. Thus, differences in resident stromal properties and tumor-derived cues may collectively determine the extent and functional organization of the CAF compartment across tumor types.

### Functional states and plasticity of CAFs

**Fig. 1:**
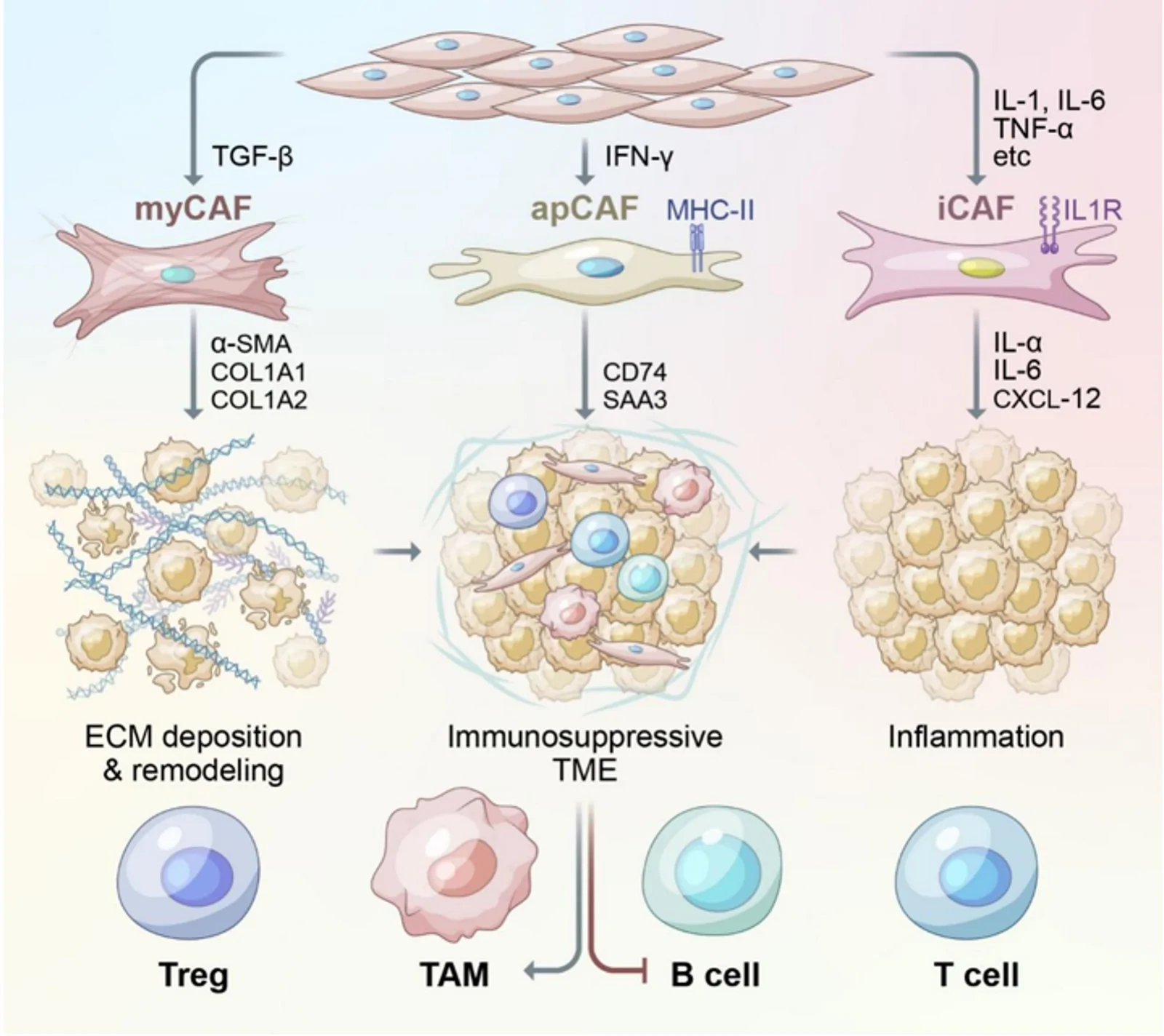
The subtypes, activating factors, markers and functions of CAFs. Under the influence of factors such as TGF-β, IFN-γ, and IL-1α, CAFs undergo phenotypic transformation into myCAFs, apCAFs, and iCAFs. MyCAFs are associated with the remodeling and deposition of the ECM, apCAFs are involved in antigen presentation, and iCAFs are related to imflammation. Each of these subtypes may contribute to the formation of an immunosuppressive TME through distinct mechanisms

Recent single-cell RNA sequencing (scRNA-seq) studies have revealed increasing complexity within CAF populations, leading to the identification of multiple CAF states with distinct phenotypic and functional characteristics. Among the most frequently described are myofibroblastic CAFs (myCAFs), inflammatory CAFs (iCAFs), and antigen-presenting CAFs (apCAFs), which have provided a useful framework for understanding CAF heterogeneity and function (Fig. 1) [13].

myCAFs are generally characterized by elevated expression of contractile and ECM-related genes, including ACTA2, TAGLN, and COL1A1 [51, 52]. Their differentiation is largely driven by transforming growth factor-β (TGF-β) signaling, with additional regulation by mechanotransduction pathways such as YAP/TAZ [53]. Functionally, myCAFs are major contributors to ECM deposition and stromal remodeling. Through the production and remodeling of matrix components, particularly collagens, they promote tissue stiffening and the formation of desmoplastic barriers [54]. These structural alterations may restrict immune-cell infiltration and influence therapeutic responses [55].

Compared with myCAFs, iCAFs are characterized by robust secretion of inflammatory cytokines and chemokines, including IL-6, IL-8, CXCL1, and CXCL12, reflecting their prominent immunomodulatory capacity [30]. Their differentiation is largely driven by IL1α-mediated activation of NF-κB and JAK/STAT signaling pathways, whereas TGF-β signaling favors the myCAF program [56, 57]. Through the secretion of cytokines and chemokines, iCAFs actively shape the immune microenvironment by modulating the recruitment and function of diverse immune cell populations [58].

In addition to myCAFs and iCAFs, apCAFs have emerged as another frequently described CAF state. These cells express high levels of MHC class II-related molecules, including HLA-DRA, HLA-DRB1, and CD74, but generally lack the classical co-stimulatory molecules required for effective T-cell activation [59]. The developmental origins and immune functions of apCAFs vary across tumor contexts. In PDAC, mesothelial cells undergo an IL-1- and TGF-β-driven transition into apCAFs, losing mesothelial identity while acquiring fibroblastic features. These mesothelial cell-derived apCAFs directly engage naive CD4^+^ T cells and drive their antigen-specific differentiation into regulatory T cells (Tregs), supporting an immunosuppressive microenvironment [60]. A recent study further showed that α-SMA⁺ CAFs can internalize and process tumor antigens and form immunological synapses with Foxp3⁺ Tregs, promoting their antigen-specific activation and proliferation. Disruption of autophagy in α-SMA⁺ CAFs reduced Treg infiltration and restrained tumor development, demonstrating the functional importance of autophagy in this interaction [61]. Cytokine signaling further shapes apCAF abundance and immunoregulatory function. In non-small cell lung cancer (NSCLC), interferon-γ (IFN-γ) drives apCAF expansion through JAK1/2-STAT1-IFI6/IFI27 signaling. In this context, PD-L2 on apCAFs promotes the accumulation of FOXP1⁺ Tregs by engaging RGMB [62]. Overall, these findings highlight the active role of apCAFs in shaping Treg responses through antigen presentation and cytokine-dependent signaling.

However, the growing number of reported CAF states has highlighted the limitations of current classification systems. The absence of definitive molecular markers and the considerable phenotypic overlap among CAF states make it difficult to establish discrete and universally applicable CAF subtypes. To address this issue, Sahai et al. proposed a conceptual framework that describes CAFs according to their dominant biological functions rather than predefined subtype labels. In this framework, CAFs are categorized based on activities such as matrix remodeling, inflammatory signaling, immune regulation, and vascular interactions, providing a more flexible approach to interpreting CAF heterogeneity across studies [16].

Importantly, accumulating evidence suggests that myCAFs, iCAFs, and apCAFs do not represent rigidly separated populations. Rather, CAF phenotypes appear to exist along a functional continuum, with substantial overlap among transcriptional programs and varying degrees of plasticity across states [63]. Spatially, distinct CAF states are frequently associated with specific microenvironmental niches. myCAFs are typically enriched adjacent to malignant cells or at the invasive tumor interface, whereas iCAFs are more commonly found in distal stromal regions and may colocalize with infiltrating immune cells [64, 65]. Distinct CAF states also contribute to the organization of different immune niches within the TME. In head and neck squamous cell carcinoma (HNSCC), IL-11^+^ iCAFs are spatially associated with inflammatory monocytes [66]. In hepatocellular carcinoma (HCC), FAP⁺ CAFs localize near CD8⁺ T cells expressing programmed cell death protein 1 (PD-1) within an intratumoral inflammatory niche, whereas CAF-C7 cells colocalize with SPP1⁺ macrophages in a distinct marginal wound-healing niche [67]. In gastric cancer, apCAFs are preferentially located near tertiary lymphoid structures (TLSs) and have been shown to enhance local T-cell activation, suggesting that their spatial positioning may support antitumor immune responses [68]. Additionally, local environmental conditions may, in turn, shape both the spatial distribution and phenotypic state of CAFs. In PDAC, iCAFs preferentially accumulate in hypoxic stromal regions distant from blood vessels [69], and hypoxia cooperates with cancer cell-derived cytokines to promote the iCAF program through hypoxia-inducible factor 1α (HIF-1α)-dependent signaling [70]. Taken together, these findings indicate that CAF states are spatially organized by local cellular, inflammatory, and metabolic cues rather than being randomly distributed throughout the tumor stroma.

CAF states are dynamically remodeled in response to evolving microenvironmental conditions. TGF-β and IL-1 signaling exert opposing effects on myCAF and iCAF programs, thereby shaping the balance and transitions between these states [30]. Experimental models further demonstrate that myCAF and iCAF phenotypes are reversible. In a three-dimensional co-culture system, pancreatic stellate cells exposed to tumor organoids or their conditioned medium acquired an iCAF phenotype, whereas two-dimensional plastic culture favored myCAF differentiation. Switching the culture conditions reversed these states: iCAFs rapidly became myCAF-like in monolayer culture, while myCAFs reverted to a quiescent state in three-dimensional matrix and could subsequently be induced into iCAFs by tumor-derived signals [64]. Metabolic cues can also contribute to CAF phenotypic remodeling. In PDAC, glutamine deprivation activates a MEK-ERK-dependent inflammatory program in myCAFs, whereas macropinocytosis enables extracellular protein scavenging and preserves the myCAF state under nutrient-limited conditions. Disruption of macropinocytosis consequently promotes myCAF-to-iCAF conversion and remodels the stromal architecture in vivo [71].

### Functional duality of CAFs

CAF heterogeneity and plasticity also challenge the traditional view of CAFs as uniformly tumor-promoting stromal cells. Instead, CAFs can either support or restrain tumor progression and may suppress or support antitumor immunity depending on their functional state and biological context. Tumor-promoting CAFs enhance malignant progression by remodeling the ECM, supporting tumor metabolism and angiogenesis, promoting invasion and metastasis, and contributing to therapy resistance. For example, PYCR1-dependent proline synthesis in CAFs supports collagen-rich ECM deposition, thereby promoting tumor growth and metastasis [72]. In colorectal cancer, COL8A1 secreted by THBS2^+^ CAFs directly interacts with ITGB1 on oxaliplatin-resistant malignant cells, activating the PI3K/AKT signaling pathway and promoting EMT-associated oxaliplatin resistance [73]. Similarly, in NSCLC brain metastases, brain metastasis-associated CAFs promote tumor progression and therapeutic resistance through IL26- and CX3CL1-mediated signaling [74]. In addition, TGF-β-dependent LRRC15^+^ CAFs have been shown to promote tumor growth, directly suppress CD8^+^ T cell function, and limit responsiveness to ICB, illustrating how tumor-promoting CAFs can impair antitumor immunity [75]. Notably, opposing CAF functions can be observed even within the same tumor type. In pancreatic cancer, FAP^+^ CAFs have been identified as tumor-promoting, whereas α-SMA^+^ CAFs exhibit tumor-restraining activity; consistently, depletion of FAP^+^ CAFs improves survival, whereas depletion of α-SMA^+^ CAFs reduces survival [76].

By contrast, some CAF populations can restrain tumor progression or be therapeutically reprogrammed to support antitumor immunity. In pancreatic cancer, genetic deletion of type I collagen in α-SMA^+^ myofibroblasts accelerates tumor progression, accompanied by increased CXCL5-mediated recruitment of myeloid-derived suppressor cells (MDSCs) and reduced infiltration of activated CD8^+^ T cells [77]. Within the same disease context, CD105 expression distinguishes fibroblast lineages with opposing functions: CD105^+^ fibroblasts are permissive for tumor growth, whereas CD105^−^ fibroblasts suppress tumor growth in an adaptive immunity-dependent manner [78]. Consistent with the tumor-promoting role of this population, CD105⁺ CAF-derived exosomal circAMPK1 induces autophagy in pancreatic cancer cells, thereby promoting their proliferation, invasion, and metastasis [79]. In prostate cancer, YAP1 inhibition reprograms CAFs from a protumorigenic, ECM-producing phenotype toward a tumor-suppressive state, thereby enhancing antitumor immunity and responsiveness to ICB [80].

These seemingly contradictory findings do not necessarily represent an intrinsic conflict in CAF biology. Rather, they reflect differences in CAF lineage, activation state, spatial localization, tumor stage, and experimental context, as well as the limited specificity of individual CAF markers. Therefore, CAF-targeted strategies should selectively suppress or reprogram tumor-promoting CAF states rather than indiscriminately depleting the entire fibroblast compartment.

## Mechanisms driving CAF metabolic reprogramming in the TME

### Signaling mediated by soluble factors and extracellular vesicles

**Fig. 2:**
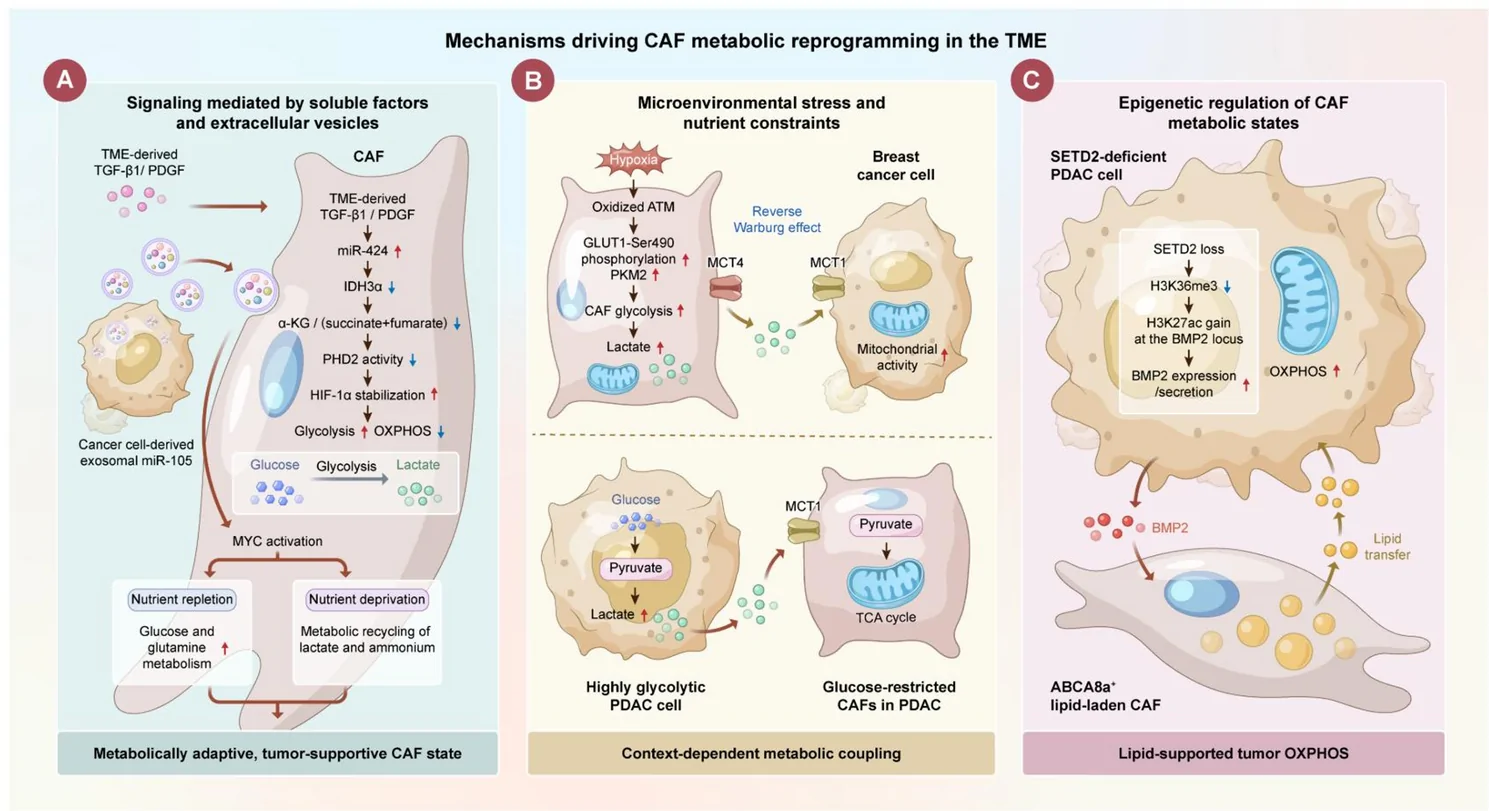
Mechanisms driving CAF metabolic reprogramming in the TME. **A** TME-derived soluble factors and tumor-cell-derived extracellular vesicles reprogram CAF metabolism through TGF-β1/PDGF-induced IDH3α downregulation and exosomal miR-105-mediated MYC activation, respectively. **B** Hypoxia and nutrient competition drive context-dependent lactate exchange between CAFs and cancer cells. CAF-derived lactate supports breast cancer-cell mitochondrial activity, whereas PDAC-derived lactate fuels the TCA cycle in glucose-restricted CAFs. **C** SETD2 loss in PDAC cells activates BMP2 signaling and induces ABCA8a⁺ lipid-laden CAFs, which supply lipids to support tumor-cell OXPHOS

Extrinsic signals within the TME are pivotal upstream drivers of CAF metabolic reprogramming. Beyond promoting fibroblast activation, these signals reshape CAF energy production and nutrient utilization. Soluble cytokines and growth factors constitute one major class of such signals. TGF-β1 and platelet-derived growth factor (PDGF) can drive CAF metabolic reprogramming from oxidative phosphorylation toward aerobic glycolysis. This transition is partly mediated by miR-424-dependent downregulation of isocitrate dehydrogenase 3α (IDH3α), which suppresses prolyl hydroxylase domain protein 2 (PHD2) activity and stabilizes HIF-1α, thereby promoting glycolytic gene expression while suppressing mitochondrial respiration (Fig. 2A) [81]. In addition to soluble mediators, CAF metabolism is also shaped by tumor-derived extracellular vesicles (EVs). Cancer-cell-derived exosomal miR-105 induces MYC-dependent metabolic reprogramming in stromal fibroblasts. This reprogramming enhances glucose and glutamine metabolism when nutrients are abundant, while enabling CAFs to recycle accumulated lactate and ammonium under nutrient-deprived conditions (Fig. 2A) [82]. A related vesicle-mediated mechanism has been reported in triple-negative breast cancer (TNBC), in which cancer-cell-derived exosomes deliver integrin β4 (ITGB4) protein to CAFs, leading to BCL2-interacting protein 3-like (BNIP3L)-dependent mitophagy and lactate production [83]. Together, these findings indicate that soluble and vesicular signals within the TME can directly induce CAF metabolic reprogramming, providing a mechanistic basis for the emergence of metabolically adapted CAF states with immune-regulatory potential.

### Microenvironmental stress and nutrient constraints

Beyond soluble and vesicular signals, local microenvironmental constraints, including nutrient depletion, hypoxia, lactate accumulation, and oxidative stress, can further drive CAF metabolic reprogramming. The reverse Warburg effect provides a classic model of tumor-induced CAF metabolic reprogramming, in which cancer cells induce aerobic glycolysis in neighboring stromal fibroblasts, leading to the release of lactate and pyruvate that fuel mitochondrial metabolism in tumor cells [84, 85]. Subsequent studies connected this stromal glycolytic program to tumor-associated oxidative stress. In fibroblasts undergoing glycolytic reprogramming, monocarboxylate transporter 4 (MCT4) is upregulated to facilitate lactate export, and this response can be attenuated by the antioxidant N-acetylcysteine [86]. In addition to promoting lactate export, tumor-associated oxidative stress can also induce autophagy in stromal fibroblasts, including mitophagy-mediated mitochondrial clearance, thereby reducing mitochondrial dependence and reinforcing aerobic glycolysis in CAFs [87]. Tumor hypoxia can further enhance this glycolytic reprogramming. In breast cancer, hypoxia induces oxidative activation of ataxia telangiectasia mutated (ATM) kinase in stromal fibroblasts. Oxidized ATM enhances glycolysis by phosphorylating glucose transporter 1 (GLUT1) at Ser490 and increasing pyruvate kinase M2 (PKM2) expression. These changes increase lactate production and enhance metabolic coupling between CAFs and cancer cells (Fig. 2B) [88].

However, lactate exchange in the TME is not unidirectional from CAFs to cancer cells. In pancreatic cancer, highly glycolytic cancer cells consume most of the available glucose, creating glucose-restricted conditions for neighboring CAFs. Under these conditions, CAFs take up cancer-derived lactate through monocarboxylate transporter 1 (MCT1) and use it as a fuel for the tricarboxylic acid (TCA) cycle (Fig. 2B) [89]. Beyond lactate metabolism, glutamine limitation can further reshape CAF nutrient metabolism. In ovarian cancer, glutamine scarcity promotes a glutamine-synthetic program in CAFs, characterized by high glutamine synthetase activity and the use of atypical carbon and nitrogen sources for glutamine synthesis. This adaptation complements glutaminase-dependent glutamine utilization by neighboring cancer cells, thereby sustaining tumor growth under nutrient-limited conditions [90].

### Epigenetic regulation of CAF metabolic states

Metabolic reprogramming may begin as MSCs acquire a CAF phenotype, rather than occurring exclusively in established CAFs. In PDAC, tumor cell-derived lactate increases α-ketoglutarate production in MSCs, thereby activating ten-eleven translocation (TET) enzymes and leading to decreased cytosine methylation and increased hydroxymethylation during the de novo differentiation of MSCs into CAFs. Co-injection of tumor cells with TET-deficient MSCs inhibited tumor growth in vivo, demonstrating the functional relevance of TET-dependent reprogramming during CAF formation [91]. In established CAFs, epigenetic remodeling can further regulate metabolic states by altering the expression of metabolism-related genes. In breast cancer, chronic hypoxia induces DNA methylation changes in fibroblasts involving HIF-1α and glycolysis-related genes, resulting in increased lactate and pyruvate production [92]. Nicotinamide N-methyltransferase (NNMT) provides another representative example of metabolic-epigenetic coupling in CAFs. In ovarian cancer, NNMT consumes S-adenosylmethionine (SAM) and reduces histone H3 lysine 27 trimethylation (H3K27me3) in CAFs, linking methyl-donor metabolism to stromal transcriptional remodeling and CAF activation [93]. Beyond CAF-intrinsic mechanisms, tumor-cell epigenetic dysregulation can also shape metabolically specialized CAF populations. In pancreatic cancer, tumor-cell SETD2 loss reduces H3K36me3 and promotes H3K27ac-associated BMP2 signaling, driving the expansion of ABCA8a⁺ lipid-laden CAFs that supply lipids to support OXPHOS in tumor cells (Fig. 2C) [20]. More recently, gemcitabine-resistant CAFs in pancreatic cancer were shown to acquire a mitophagy-competent metabolic state. Mechanistically, SETD1A-mediated H3K4 methylation activates ZEB1 and drives this transition. This ZEB1-driven CAF program activates tumor-cell pyrimidine metabolism and increases nucleotide availability, ultimately supporting gemcitabine resistance [94]. These findings indicate that epigenetic regulation can establish distinct CAF metabolic states and enable CAFs to support tumor-cell metabolism under microenvironmental or therapeutic stress.

## CAF metabolic reprogramming orchestrates the tumor immune microenvironment

CAF metabolic reprogramming regulates the tumor immune microenvironment through several interconnected mechanisms. It can reshape immune-cell localization by reinforcing stromal barriers, selectively recruit or retain immune populations through metabolite- and cytokine-dependent signals, and alter immune-cell function through nutrient competition or metabolite accumulation [95]. In some contexts, CAFs also engage in reciprocal metabolic crosstalk with immune cells, further reprogramming their metabolic states and functional phenotypes. These processes provide a framework for understanding how metabolically distinct CAF states contribute to immune-excluded, immunosuppressive, immunoreactive, and emerging neuroimmune niches. Representative mechanisms linking CAF metabolic reprogramming to immune-excluded and immunosuppressive niches are summarized in Table 1.

**Table 1:** CAF metabolic crosstalk with immune cells across distinct immune niches

Immune niche	Metabolic feature	Immune cell	Mode of crosstalk	Key mediators	Immune consequence	Refs.
Immune-excluded niche	GLUT1-driven glycolytic reprogramming	CD8⁺ T cells	Indirect: chemokine-mediated	GLUT1; CXCL16	Peripheral retention of CD8⁺ T cells and impaired tumor infiltration	[96]
Immune-excluded niche	LINC01711-LDHA-mediated glycolytic reprogramming	CD8⁺ T cells	Indirect: metabolite-mediated	LDHA; lactate	Lactate accumulation impairs T-cell infiltration and function	[97]
Immune-excluded niche	TDO2-mediated tryptophan metabolism	CD4⁺ and CD8⁺ T cells	Indirect: metabolite/chemokine-mediated	TDO2; kynurenine; CXCL9/10/11	Peripheral sequestration of T cells, increased Treg differentiation, and CD8⁺ T-cell dysfunction	[98]
Cytotoxic lymphocyte dysfunction niche	CD73-dependent adenosine production	CD8⁺ T cells	Indirect: metabolite-mediated	CD73; adenosine; A2BR; A2AR	Extracellular adenosine suppresses CD8⁺ T-cell function	[99]
Cytotoxic lymphocyte dysfunction niche	Arginase-mediated L-arginine depletion	CD8⁺ T cells	Indirect: nutrient depletion/soluble factor-mediated	Arginase; VISTA; HVEM-BTLA	Impaired CD8⁺ T-cell activation and cytotoxicity, with arginase-dependent upregulation of TIGIT and BTLA	[100]
Cytotoxic lymphocyte dysfunction niche	PLA2G2A-mediated phospholipid remodeling	CD8⁺ T cells	Indirect: secreted enzyme-mediated	PLA2G2A	Reduced CD8⁺ T-cell activation and effector function	[101]
Immune niche	Metabolic feature	Immune cell	Mode of crosstalk	Key mediators	Immune consequence	Ref.
Cytotoxic lymphocyte dysfunction niche	NNMT-mediated metabolic and epigenetic reprogramming	CD8⁺ T cells	Indirect: secreted factor-mediated	NNMT; ANGPTL4	CD8⁺ T-cell dysfunction and reduced ICB responsiveness	[102]
Cytotoxic lymphocyte dysfunction niche	NetG1⁺ CAF-mediated glutamate/glutamine metabolic reprogramming	NK cells	Indirect: metabolite-mediated	NetG1; glutamate/glutamine	Impaired NK-cell cytotoxic function	[103]
Cytotoxic lymphocyte dysfunction niche	Impaired CAF ketogenesis	NK cells	Indirect: chemokine-mediated	HMGCS2; CXCL12	Reduced cytotoxic lymphocyte infiltration and impaired antitumor immunity	[104]
Cytotoxic lymphocyte dysfunction niche	PGE2/IDO1-mediated immunosuppressive metabolism	NK cells	Indirect: metabolite/enzyme-mediated	PGE2; IDO1	Reduced NK-cell activation and cytotoxicity	[105]
Cytotoxic lymphocyte dysfunction niche	PDPN⁺ CAF-mediated tryptophan metabolic reprogramming	NK cells	Indirect: metabolite/enzyme-mediated	IDO1;TDO2; kynurenine	Impaired NK-cell cytotoxic function	[106]
Cytotoxic lymphocyte dysfunction niche	Iron metabolic reprogramming	NK cells	Indirect: secreted factor-mediated	FSTL1-DIP2A; NCOA4	NK-cell iron accumulation, ferroptosis susceptibility, and impaired cytotoxic function	[107]
Treg-enriched niche	Glycolytic reprogramming	CD4⁺ T cells (Th1/Tregs)	Indirect: metabolite-mediated	Lactate	Reduced Th1 differentiation and increased FoxP3⁺ Treg differentiation	[108]
Immune niche	Metabolic feature	Immune cell	Mode of crosstalk	Key mediators	Immune consequence	Ref.
Myeloid suppressive niche	iCAF chemokine program driving LAM accumulation	Monocytes/LAMs	Indirect: chemokine-mediated	CXCL12-CXCR4; TREM2; STAB1	Monocyte recruitment and accumulation of immunosuppressive LAMs	[115]
Myeloid suppressive niche	Lactate-induced CAF activation and inflammatory reprogramming	Macrophages	Indirect: cytokine-mediated	Lactate; IL-8	TAM recruitment and M2-like polarization	[119]
Myeloid suppressive niche	HMOX1-driven iron accumulation and epigenetic reprogramming	Immunosuppressive myeloid cells	Indirect: secreted factor-mediated	HMOX1; KDM6B; CCL2; CSF1; CXCL1	Recruitment of immunosuppressive myeloid populations	[124]
Myeloid suppressive niche	Amyloid-β-associated redox signaling	Neutrophils	Indirect: ligand-receptor-mediated	Amyloid β-CD11b; ROS-PAD4	NET formation and reciprocal CAF matrix remodeling	[131]
Myeloid suppressive niche	Bile acid-induced chemokine program	Neutrophils	Indirect: chemokine-mediated	Bile acid-GPBAR1 signaling; CXCL10	Neutrophil recruitment and formation of an immunosuppressive niche	[133]
Antigen-presentation-impaired niche	IL-6-driven tryptophan metabolic reprogramming	Monocyte-derived DCs	Indirect: cytokine-mediated	IL-6; IDO; kynurenine	Impaired DC antigen presentation and T-cell priming, with Treg expansion	[140]

### Immune-excluded niches

**Fig. 3:**
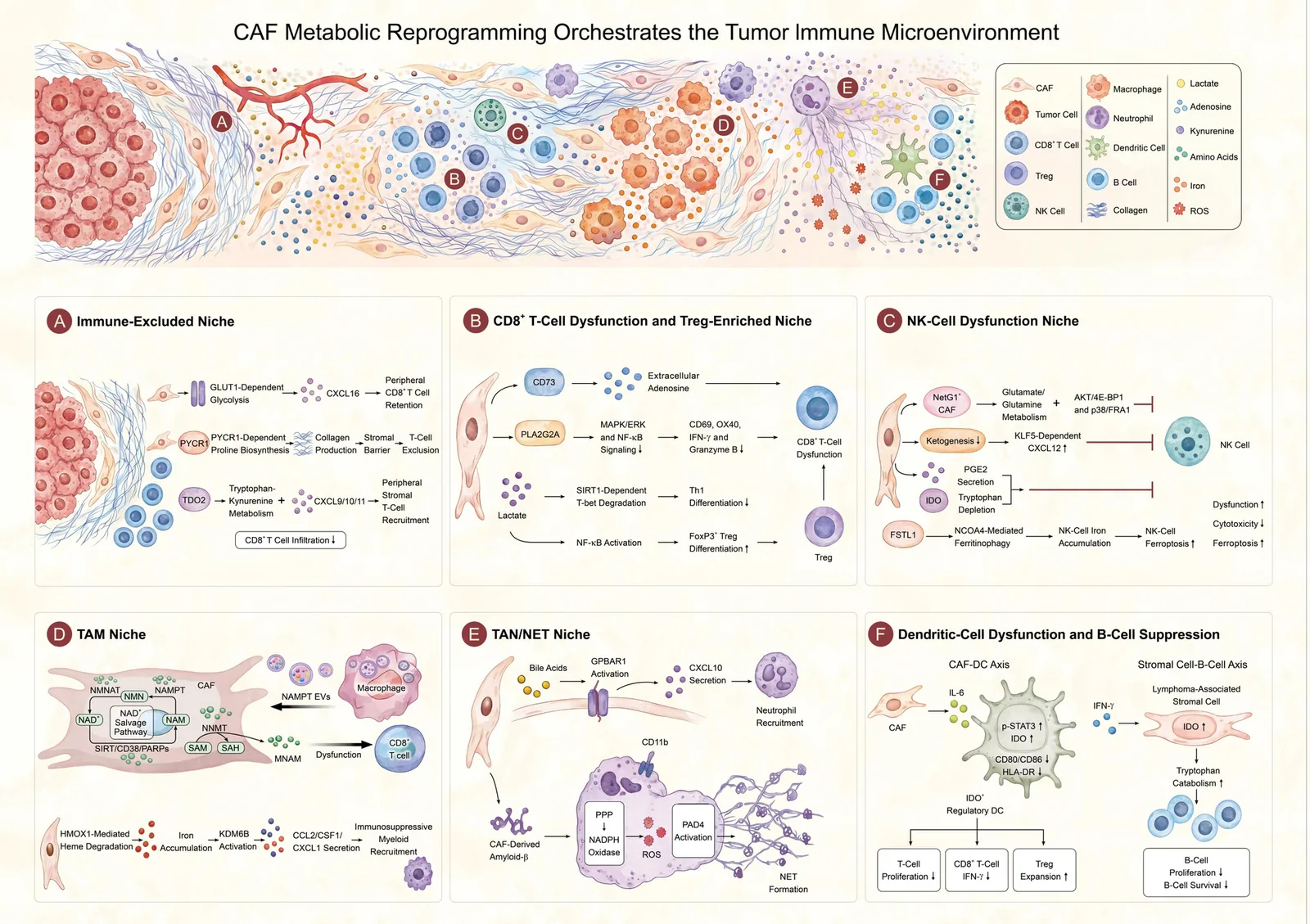
CAF metabolic reprogramming shapes distinct immune niches in the TME. **A** GLUT1-dependent glycolysis, PYCR1-dependent proline biosynthesis, and TDO2-associated tryptophan-kynurenine metabolism promote stromal T-cell retention and exclusion. **B** CAF-derived adenosine, PLA2G2A, and lactate impair CD8⁺ T-cell function and promote Treg enrichment. **C** Altered glutamate/glutamine metabolism, reduced ketogenesis, PGE₂ secretion, tryptophan depletion, and FSTL1-induced ferritinophagy contribute to NK-cell dysfunction and ferroptosis. **D** The NAMPT-NNMT nicotinamide metabolic circuit and the HMOX1-iron-KDM6B axis regulate T-cell dysfunction and immunosuppressive myeloid-cell recruitment. **E** Bile acid-GPBAR1-CXCL10 signaling promotes neutrophil recruitment, whereas CAF-derived amyloid-β induces ROS- and PAD4-dependent NET formation. (**F**) IL-6-STAT3-IDO signaling induces regulatory DCs, whereas stromal IDO-dependent tryptophan catabolism suppresses B-cell proliferation and survival

**Fig. 4:**
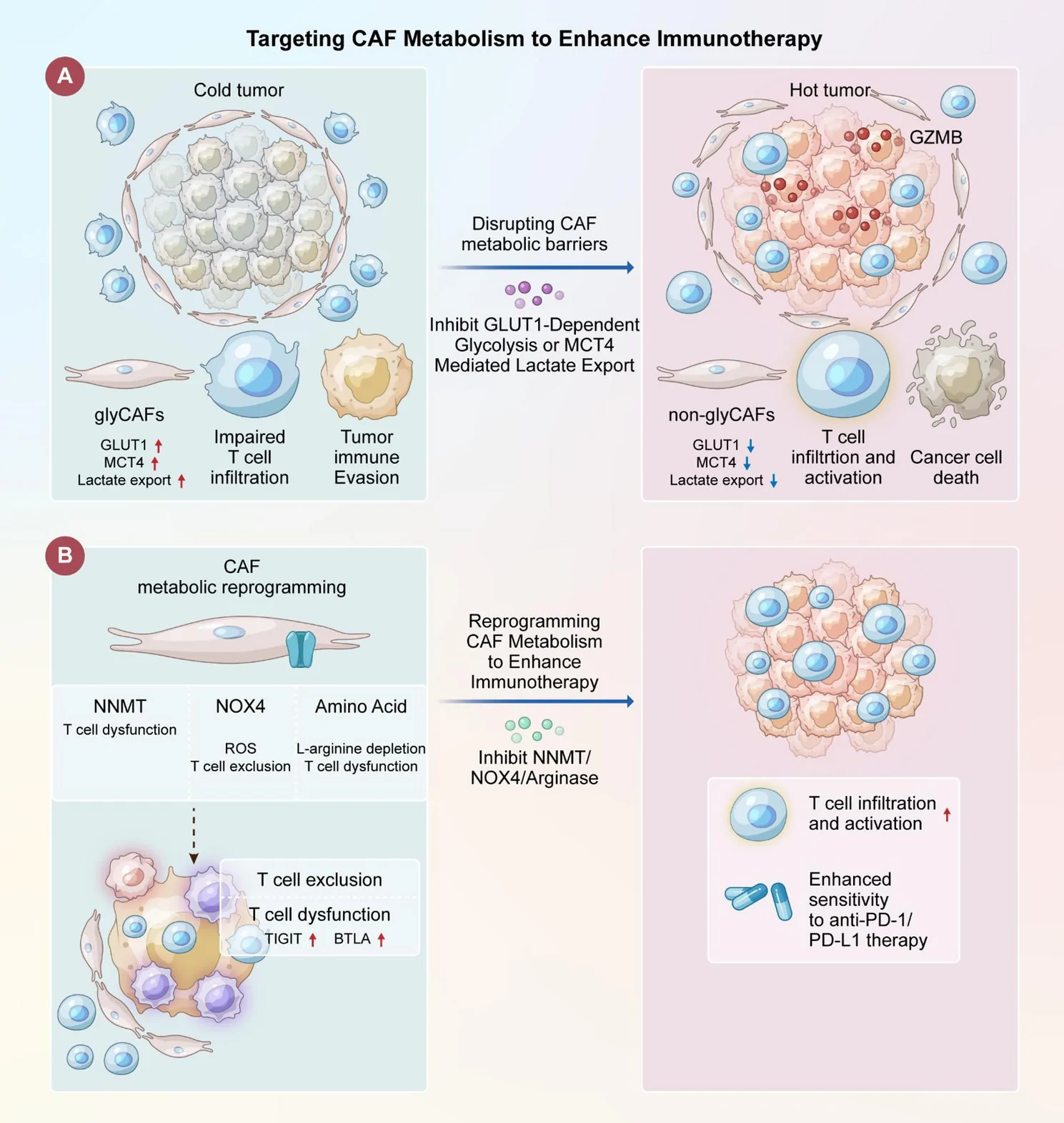
Targeting CAF metabolism to enhance immunotherapy. **A** Glycolytic CAFs establish a lactate-enriched, immune-excluded microenvironment through GLUT1-dependent glycolysis and MCT4-mediated lactate export. Targeting these metabolic barriers promotes CD8⁺ T-cell infiltration and activation, facilitating the conversion of “cold” tumors into “hot” tumors. **B** Targeting CAF-associated metabolic and redox programs, including NNMT, NOX4, and arginase-mediated L-arginine depletion, alleviates T-cell exclusion and dysfunction and enhances responsiveness to immune checkpoint blockade

Immune-excluded niches are typically marked by the retention of cytotoxic CD8⁺ T cells at stromal borders or tumor margins, with limited penetration into tumor nests. CAF metabolic reprogramming can promote this spatial pattern by reinforcing stromal barriers. These barriers may be metabolic, involving localized metabolite accumulation or nutrient imbalance; structural, involving collagen deposition, matrix cross-linking, and stromal stiffening; or chemokine-guided, attracting T cells to peripheral regions while limiting effective intratumoral infiltration. In soft tissue sarcoma, CAFs accumulate at the tumor margin and rely on GLUT1-dependent glycolysis to promote CXCL16 secretion, thereby impeding CD8⁺ T-cell entry into the tumor core. Pharmacological inhibition of GLUT1 with BAY-876 reduced CAF glycolytic activity, decreased CXCL16 secretion, restored T-cell infiltration, and enhanced chemotherapy sensitivity (Figs. 3A and 4A) [96]. CAF metabolism can also restrict T-cell entry by reshaping the local metabolite milieu. FAP⁺ CAFs overexpress LINC01711, which enhances lactate dehydrogenase A (LDHA) activity and glycolytic flux, leading to increased MCT4-dependent lactate export. The resulting lactate-rich niche forms a metabolic barrier that impedes CD8⁺ T-cell infiltration and function, whereas silencing LINC01711 restores antitumor immunity and sensitizes tumors to anti-PD-1 therapy (Fig. 4A) [97]. Structural barriers may also arise from CAF metabolic programs that support ECM deposition. PYCR1-dependent proline biosynthesis promotes collagen production in CAFs, potentially contributing to matrix-rich stromal regions that restrict T-cell entry into tumor nests (Fig. 3A) [72]. Beyond matrix remodeling, CAF metabolic reprogramming also influences T-cell positioning by reshaping chemokine secretion. In oral squamous cell carcinoma (OSCC), CAFs expressing tryptophan 2,3-dioxygenase 2 (TDO2) enhance tryptophan-kynurenine metabolism and secrete CXCL9, CXCL10, and CXCL11, thereby recruiting T cells to peripheral stromal regions while limiting their penetration into tumor nests (Fig. 3A) [98]. These studies indicate that CAF metabolic programs regulate antitumor immunity by shaping T-cell infiltration and highlight CAF metabolism as a potential target for enhancing ICB responsiveness.

### Immunosuppressive niches

Within immunosuppressive niches, antitumor immune cells are not necessarily excluded from tumor tissue but are rendered functionally ineffective. These regions are typically characterized by impaired effector CD8⁺ T-cell activity, enrichment of Tregs, tumor-associated macrophages (TAMs), and tumor-associated neutrophils (TANs), together with dysfunctional dendritic cells (DCs) and other suppressive stromal or immune populations. CAF metabolic reprogramming can contribute to this state by reshaping the local metabolite milieu, cytokine and chemokine networks, redox balance, and stromal-immune interactions. These effects can be conceptualized as two interrelated mechanisms: impairment of effector immune-cell activity and remodeling of suppressive immune-cell composition within the TME.

#### Cytotoxic lymphocyte dysfunction and Treg-enriched niche

The accumulation of immunosuppressive metabolites in the TME represents a key mechanism by which CAF metabolic reprogramming impairs effector T-cell function. Among these metabolites, purinergic mediators such as adenosine have received particular attention. CAFs are a major non-immune source of ecto-5′-nucleotidase (CD73) in the TME. Yu et al. [99] demonstrated that CD73 expression in CAFs is regulated by adenosine A2B receptor (A2BR) signaling, forming a positive feedback loop that sustains extracellular adenosine accumulation and suppresses CD8⁺ T-cell function. Pharmacological blockade of CD73 or A2BR disrupts this axis, restoring T-cell infiltration and limiting tumor progression (Fig. 3B). In addition, sustained nutrient competition within the TME may promote T-cell dysfunction. Melanoma-associated fibroblasts can restrict T-cell function through arginase-mediated L-arginine depletion. In CD8⁺ T cells, this metabolic constraint limits mTOR-dependent protein synthesis and attenuates ERK and NF-κB signaling, resulting in reduced CD69 expression and impaired cytotoxic effector function, including granzyme B production. Sustained metabolic stress further promotes the upregulation of inhibitory receptors such as TIGIT and BTLA on CD8⁺ T cells, while fibroblast-expressed immunomodulatory ligands including VISTA and HVEM reinforce this suppressive state (Fig. 4B) [100].

CAF lipid-associated inflammatory signaling also contributes to CD8⁺ T-cell functional impairment. In pancreatic cancer, PLA2G2A⁺ CAFs secrete high levels of phospholipase A2 group IIA (PLA2G2A), a phospholipid-modifying enzyme that suppresses MAPK/ERK and NF-κB signaling in CD8⁺ T cells. Notably, despite increased CD8⁺ T-cell infiltration in PLA2G2A⁺ CAF-enriched tumors, these cells exhibit a dysfunctional phenotype characterized by reduced CD69 and OX40 expression, along with diminished IFN-γ and granzyme B production (Fig. 3B) [101]. In addition, CAF metabolic reprogramming has been linked to epigenetic mechanisms that shape T-cell dysfunction. CAF-expressed NNMT promotes tumor immune evasion through enhancer of zeste homolog 2 (EZH2)-dependent epigenetic reprogramming and angiopoietin-like 4 (ANGPTL4)-driven metabolic-immune coupling, resulting in CD8⁺ T-cell suppression. Targeting the NNMT-ANGPTL4 axis restores cytotoxic T-cell activity and enhances responsiveness to ICB (Fig. 4B) [102].

Beyond CD8⁺ T cells, CAF metabolic reprogramming can impair the function of other cytotoxic lymphocyte populations, including natural killer (NK) cells. In pancreatic cancer, NetG1^+^ CAFs establish an immunosuppressive metabolic niche by regulating glutamate/glutamine metabolism, supporting tumor survival while suppressing NK cell-mediated cytotoxicity through a NetG1-dependent signaling program involving AKT/4E-BP1 and p38/FRA1 pathways (Fig. 3C) [103]. CAF metabolic reprogramming can also shape cytotoxic lymphocyte function through chemokine remodeling. Impaired ketogenesis in CAFs promotes KLF5-dependent CXCL12 production and restricts NK-cell and cytotoxic T-cell responses, contributing to an immunosuppressive TME. Restoration of CAF ketogenesis reduces CXCL12 expression, enhances antitumor immune-cell infiltration, and sensitizes tumors to anti-PD-1 therapy [104].

CAFs can also suppress NK-cell function through metabolically regulated soluble mediators. In HCC, CAFs impair NK-cell activation and cytotoxicity through prostaglandin E2 (PGE2) secretion and indoleamine 2,3-dioxygenase (IDO)-mediated tryptophan catabolism (Fig. 3C) [105]. Similarly, podoplanin-positive (PDPN⁺) CAFs in human epidermal growth factor receptor 2 (HER2)-positive breast cancer promote trastuzumab resistance by expressing the immunosuppressive enzymes indoleamine 2,3-dioxygenase 1 (IDO1) and tryptophan 2,3-dioxygenase 2 (TDO2), which suppress NK-cell-mediated antibody-dependent cellular cytotoxicity [106]. Altered iron homeostasis in CAFs has recently been linked to cytotoxic lymphocyte dysfunction. In gastric cancer, CAFs remodel iron metabolism and promote iron accumulation in NK cells, with increased expression of the iron-regulatory genes ferroportin 1 and hephaestin. Moreover, CAF-derived follistatin-like protein 1 (FSTL1) induces nuclear receptor coactivator 4 (NCOA4)-mediated ferritinophagy through the DIP2A-p38 MAPK pathway, increasing NK-cell susceptibility to ferroptosis and impairing cytotoxic activity (Fig. 3C) [107].

CAF metabolic reprogramming not only impairs cytotoxic lymphocyte function but also promotes the establishment of suppressive immune niches within the TME. Among suppressive immune populations, Tregs represent a plausible but still underexplored target of CAF-driven metabolic immunosuppression. In prostate cancer, CAF-derived lactate reshapes CD4⁺ T-cell polarization by suppressing T helper 1 (Th1) differentiation through sirtuin 1 (SIRT1)-dependent T-bet degradation and promoting FoxP3⁺ Treg differentiation via NF-κB activation. This process establishes a Treg-enriched immunosuppressive milieu that supports tumor progression (Fig. 3B) [108]. These observations suggest that CAF metabolic reprogramming may create metabolically selective niches that favor Treg persistence and function while limiting effector T-cell activity. However, the extent to which distinct CAF metabolic states directly control Treg recruitment, metabolic adaptation, and suppressive function remains poorly resolved.

#### TAM- and MDSC-associated immunosuppressive niches

TAMs are abundant and metabolically plastic immune cells in the TME [109]. Although historically classified as pro-inflammatory M1 or anti-inflammatory M2 macrophages [110, 111], this binary framework is now considered overly simplistic, as TAMs display substantial functional and metabolic heterogeneity across tumor types and disease stages [112, 113]. Current classification frameworks increasingly emphasize functionally defined TAM states, including lipid-associated, interferon-responsive, antigen-presenting, inflammatory, and immunosuppressive macrophage programs [114–118]. However, direct evidence linking CAF metabolic reprogramming to interferon-responsive or antigen-presenting TAM states remains limited, with current mechanistic studies focusing predominantly on lipid-associated and immunosuppressive macrophage programs. In this context, CAF metabolic reprogramming may shape specific TAM programs rather than simply shifting macrophages toward a generic M2-like state.

Lactate provides a well-defined example of how CAF metabolic reprogramming can redirect TAM function. In lung cancer, tumor cell-derived lactate promotes NUSAP1 nuclear translocation in fibroblasts, facilitating the recruitment of the JUNB-FRA1-FRA2 transcriptional complex to the DESMIN promoter and increasing DESMIN transcription and CAF activation. These DESMIN⁺ CAFs secrete IL-8, which promotes TAM recruitment and favors M2-like polarization, reinforcing an immunosuppressive TME and limiting ICB efficacy [119]. Lipid-associated macrophages (LAMs) represent a distinct TAM state associated with a CAF-rich TME. In TNBC, an immunosuppressive STAB1⁺TREM2^high^ LAM subset is enriched among patients resistant to ICB. CAFs promote monocyte differentiation toward a lipid-accumulating macrophage state, impairing T-cell activation, while the iCAF-derived CXCL12-CXCR4 axis further supports monocyte recruitment and LAM accumulation [115].

CAF-macrophage metabolic crosstalk is also bidirectional. Jiang et al. [120] identified a reciprocal macrophage-CAF axis involving nicotinamide phosphoribosyltransferase (NAMPT) and NNMT, linking EV-mediated signaling and nicotinamide metabolism to anti PD-1 responsiveness. Macrophage-derived EVs deliver NAMPT to CAFs, suppressing CAF NNMT expression through SIRT1-NOTCH signaling and reducing N^1^-methylnicotinamide (MNAM) production. Functionally, this macrophage-CAF metabolic circuit enhances antitumor T-cell activity and improves responsiveness to anti-PD-1 therapy (Fig. 3D). Beyond this reciprocal metabolic circuit, CAF-intrinsic NNMT also promotes immunosuppressive myeloid-cell recruitment through epigenetic reprogramming. In urothelial bladder cancer, elevated NNMT expression in CAFs was associated with resistance to PD-L1 blockade and promoted TAM recruitment by increasing serum amyloid A expression and secretion. In murine models, pharmacological NNMT inhibition with 5-amino-1-methylquinolinium iodide reduced macrophage infiltration and tumor growth and enhanced the antitumor efficacy of anti-PD-L1 therapy [121]. MDSCs comprise a heterogeneous population of immature myeloid cells that suppress antitumor immunity, facilitate tumor immune evasion, and contribute to resistance to immunotherapy. Accordingly, targeting MDSCs represents a promising strategy for improving therapeutic responses [122, 123]. In high-grade serous ovarian cancer, NNMT-mediated loss of H3K27me3 increases complement secretion by CAFs, promoting the recruitment of immunosuppressive MDSCs. Genetic or pharmacological NNMT inhibition reduces MDSC infiltration, enhances CD8⁺ T-cell activity, and restores ICB efficacy across multiple murine tumor models [22].

Iron metabolism has emerged as an important metabolic program associated with CAF-mediated myeloid immunosuppression. In prostate cancer, iron-loaded CAFs accumulate intracellular iron through heme oxygenase 1 (HMOX1)-mediated heme degradation and acquire immunosuppressive properties. This iron accumulation activates the iron-dependent epigenetic enzyme lysine demethylase 6B (KDM6B), promoting the transcription of myeloid-associated factors, including CCL2, CSF1, and CXCL1, which recruit immunosuppressive myeloid populations. Targeting the HMOX1-iron-KDM6B axis reverses the immunosuppressive phenotype of iron-loaded CAFs and restores antitumor immunity (Fig. 3D) [124]. Recent evidence further indicates that iron-loaded CAFs acquire ferroptosis resistance to maintain this immunosuppressive state. ZEB1 transcriptionally upregulates solute carrier family 7 member 11 (SLC7A11) and enhances cystine/glutamate antiporter system X⁻ activity, protecting iron-loaded CAFs from ferroptosis and maintaining their immunosuppressive phenotype [125].

#### TAN-mediated immunosuppression

Tumor-associated neutrophils (TANs) constitute a heterogeneous and highly plastic myeloid population within the TME. Although their phenotypes have traditionally been described using the pro-inflammatory N1-like and immunosuppressive N2-like framework, TANs exhibit a broad continuum of functional states shaped by tumor- and stroma-derived signals [126–128]. The acquisition of these diverse functional states is closely linked to metabolic adaptation. In particular, effector programs such as reactive oxygen species (ROS) production and certain forms of neutrophil extracellular trap (NET) formation rely on reduced nicotinamide adenine dinucleotide phosphate (NADPH) availability and metabolic flux through the pentose phosphate pathway (PPP).

Within the metabolic-inflammatory landscape of the TME, CAFs regulate neutrophil recruitment and pro-tumor functional programming, creating conditions that favor NET formation. In pancreatic cancer, CAFs promote neutrophil migration and survival, increasing neutrophil-derived IL-8 secretion, which subsequently enhances pancreatic cancer cell migration and invasion [129]. CAFs can also drive neutrophil recruitment through indirect, tumor cell-mediated inflammatory circuits. In HNSCC, CAF-associated GDF15-GFRAL signaling activates the PI3K/AKT/STAT3 pathway in tumor cells, inducing TNF-α release and oxidative stress, which in turn promotes neutrophil infiltration and pro-tumor polarization [130]. Beyond recruitment, CAFs directly regulate neutrophil effector programs, particularly NET formation. Specifically, CAF-secreted amyloid-β induces ROS-PAD4-dependent NET formation by engaging CD11b on neutrophils. CAF-rich regions are preferential sites for NET accumulation. NETs, in turn, promote CAF-mediated matrix remodeling, indicating bidirectional crosstalk between neutrophils and CAFs (Fig. 3E) [131]. Importantly, NETs generated within CAF-enriched niches actively contribute to the establishment of an immunosuppressive TME. Clinically, NET abundance is associated with poor response to ICB in non-small cell lung cancer. The close spatial association between CAFs and NET-rich regions further implicates the organization of stromal-immune niches in immunotherapy resistance [132]. In cholangiocarcinoma, elevated bile acid levels activate GPBAR1 signaling in CAFs, driving chemokine reprogramming characterized by CXCL10 upregulation, which promotes neutrophil recruitment and contributes to the establishment of an immunosuppressive TME (Fig. 3E) [133]. Furthermore, CAF-induced IL-6-STAT3 signaling promotes PD-L1 expression on neutrophils in HCC, reinforcing neutrophil-mediated immunosuppression [134]. Beyond local immunosuppression, NETs also participate in pre-metastatic niche formation, as illustrated by SPP1-driven neutrophil recruitment and NET formation during the pulmonary metastasis of HCC [135].

In summary, CAFs orchestrate neutrophil infiltration, NET formation, and immunosuppressive polarization within the TME. These programs are closely linked to metabolic processes governing redox homeostasis. However, the metabolic determinants of CAF-TAN crosstalk remain incompletely defined, particularly with respect to the roles of CAF-derived metabolites in driving neutrophil functional reprogramming.

#### Dendritic-cell dysfunction and impaired antigen presentation

Dendritic cells (DCs) are professional antigen-presenting cells that initiate adaptive immune responses by activating CD8⁺ and CD4⁺ T cells through MHC-I/II–restricted antigen presentation and costimulatory signaling [136–138]. However, within the TME, DC maturation and antigen-presenting capacity are frequently impaired, leading to insufficient T-cell priming and dysfunctional antitumor immunity [139]. As major stromal constituents, CAFs are emerging as critical regulators of DC differentiation and function. In HCC, CAFs recruit monocyte-derived DCs and promote their differentiation into IDO^+^ regulatory DCs characterized by impaired costimulatory molecule expression and reduced T-cell stimulatory capacity, facilitating Treg expansion. Mechanistically, CAF-derived IL-6 activates STAT3 signaling in DCs, driving IDO upregulation and linking CAF-DC crosstalk to the immunosuppressive tryptophan-kynurenine metabolic pathway. This process ultimately impairs T-cell priming and promotes immune evasion (Fig. 3F) [140]. Similarly, in oesophageal squamous cell carcinoma, CAF-derived WNT2 suppresses DC differentiation and maturation through SOCS3-mediated inhibition of JAK2/STAT3 signaling, thereby attenuating antigen presentation and restricting CD8⁺ T-cell infiltration. Notably, targeting WNT2 signaling restores DC function and enhances the response to anti-PD-1 therapy [141].

Beyond directly suppressing DC function, CAFs can regulate DC recruitment and spatial organization within the TME under specific conditions. In HNSCC [142], tumor-conditioned medium preferentially promoted the migration of conventional dendritic cells (cDCs) rather than T cells, and CAFs were identified as a major source of cDC-recruiting chemokines. Fibroblast depletion markedly reduced cDC migration, suggesting that specific CAF states can shape DC localization within the TME and potentially influence antitumor immune responses. Thus, although CAFs are predominantly recognized as immunosuppressive stromal components, certain CAF populations may also contribute to immune activation by promoting cDC recruitment.

#### B cell functional reprogramming niche

B cells are important components of the tumor immune microenvironment and exert context-dependent roles in tumor immunity. Activated B cells contribute to antitumor responses through antigen presentation, antibody production, and participation in tertiary lymphoid structures (TLS), whereas regulatory B cells (Bregs) promote immunosuppression through the secretion of immunomodulatory cytokines [143, 144]. Increasing evidence indicates that B-cell function is strongly influenced by the surrounding stromal context, particularly through stromal cell-derived signals that regulate TLS formation and organization [145, 146]. As major cellular components of the tumor stroma, CAFs have emerged as important regulators of B-cell localization and functional organization within the TME.

In HNSCC, FRC-like CAFs are enriched in immune-active regions and contribute to TLS formation by establishing specialized stromal niches that support B-cell recruitment, spatial organization, and functional maturation [66]. Spatial transcriptomic analyses of penile squamous cell carcinoma identified CCL21⁺ CAFs as a specialized stromal subset enriched in mature TLS regions. These CAFs were associated with B-cell recruitment through the CCL21-CCR7 axis and with germinal center-like maturation, highlighting the role of CAF heterogeneity in shaping adaptive immune niches within the TME [147]. Beyond this spatial regulation, CAF metabolic programs may further influence B-cell activity by remodeling the local metabolic landscape. In follicular lymphoma, IFN-γ induces IDO expression in lymphoma-associated stromal cells, leading to enhanced tryptophan catabolism and IDO-dependent suppression of B cell proliferation and survival (Fig. 3F) [148]. Although cytokine-mediated signaling and TLS organization have been extensively studied, the mechanisms through which CAF-associated metabolic programs regulate B-cell responses remain incompletely understood. Defining these mechanisms will provide further insights into the metabolic regulation of adaptive immunity within the TME.

## Therapeutic strategies targeting CAFs and prospects for combination with immunotherapy

CAFs have emerged as promising therapeutic targets due to their important role in shaping the TME and influencing therapeutic responses. However, limited clinical efficacy and concerns regarding CAF heterogeneity and off-target toxicity have highlighted the need for more selective approaches. Recent advances have therefore expanded CAF-targeted therapies beyond simple depletion toward functional modulation, stromal remodeling, and metabolic intervention, with increasing interest in combining these strategies with immunotherapy. Representative conventional CAF-targeted strategies are summarized in Table 2.

**Table 2:** CAF-targeted therapeutic strategies: preclinical and clinical evidence, and combination approaches

Strategy	Target/approach	Intervention or agent	Cancer/model	Combination strategy	Key findings	Stage/safety	Ref.
FAP-directed targeting	Antibody	Sibrotuzumab (F19)	Advanced solid tumors; patients	Monotherapy	Selective localization to FAP-expressing tumor stroma, with limited clinical antitumor activity	Phase I; acceptable safety	[149]
	Enzymatic inhibition	Talabostat (Val-boroPro)	Metastatic colorectal cancer; patients	Monotherapy	Reduced circulating FAP activity, with limited disease stabilization and no objective responses	Phase II; generally well tolerated	[150]
	Vaccination	Oral FAP DNA vaccine	Murine colon and breast cancer models	Doxorubicin	Induced CD8⁺ T-cell-mediated depletion of FAP^+^ fibroblasts, reduced collagen deposition, and improved intratumoral drug uptake	Preclinical	[151]
	Vaccination	Synthetic consensus FAP DNA vaccine	Murine solid tumor models	Tumor antigen-specific vaccine	Induced FAP-specific CD4⁺ and CD8⁺ T-cell responses and enhanced antitumor immunity, although efficacy remained limited by the immunosuppressive TME	Preclinical	[152]
	Vaccination	FAP-targeted vaccine	Murine breast cancer model	Cyclophosphamide	Enhanced cytotoxic T-cell responses, reduced immunosuppressive stromal components, and improved tumor control	Preclinical	[153]
	CAR-T	FAP-specific CAR-T cells	Multiple murine solid tumor models	Monotherapy	Depleted FAP⁺ CAFs, disrupted tumor stroma, and increased CD8⁺ T-cell infiltration	Preclinical; severe bone toxicity	[154]
	CAR-T	FAP-specific CAR-T cells	Multiple murine solid tumor models	Monotherapy	Inhibited tumor growth and enhanced endogenous CD8⁺ T-cell responses	Preclinical; minimal off-tumor toxicity	[155]
	CAR-T	FAP-specific CAR-T cells	Murine pancreatic cancer model	Sequential CLDN18.2-targeted CAR-T therapy	Reduced MDSC recruitment and enhanced persistence and activity of tumor-targeted CAR-T cells	Preclinical	[156]
	CAR-T	Human/murine cross-reactive FAP-specific CAR T cells	Multiple solid tumor models	Monotherapy	Targeted FAP⁺ tumor cells and CAFs with potent antitumor activity	Preclinical; low off-tumor toxicity	[157]
	CAR-T	Intrapleural FAP-targeted CAR T cells (NCT01722149)	Malignant pleural mesothelioma; patients	Subsequent anti-PD-1 in one patient	Demonstrated feasibility and preliminary safety; antitumor efficacy remained inconclusive	Phase I; no treatment-related serious toxicity; two unrelated thromboembolic events	[158]
ECM remodeling	Collagen reduction	Losartan	Murine breast, pancreatic, and skin tumor models	Oncolytic HSV; liposomal doxorubicin	Reduced stromal collagen and improved intratumoral delivery and efficacy of oncolytic HSV and liposomal doxorubicin	Preclinical	[161]
	Collagen/HA reduction	Losartan	Murine pancreatic cancer models	Chemotherapy	Reduced stromal collagen and HA, improving perfusion and drug delivery while alleviating tumor hypoxia	Preclinical	[162]
	LOX inhibition	Genetic or pharmacologic reduction of LOX activity	Murine mammary cancer models	Monotherapy	Reduced ECM stiffening, focal adhesion formation, PI3K signaling, and tumor progression	Preclinical	[163]
	LOXL2 inhibition	AB0023, LOXL2-specific antibody	Primary and metastatic tumor xenograft models	Monotherapy	Reduced activated fibroblasts, desmoplasia, vascular remodeling, and tumor growth	Preclinical	[164]
	LOXL2 inhibition	Simtuzumab	Metastatic pancreatic adenocarcinoma; patients	Gemcitabine	Failed to improve PFS, OS, or objective response rate	Phase II; generally well tolerated	[165]
	Pan-LOX inhibition	PXS-5505	Pancreatic cancer models	Gemcitabine	Softened fibrotic stroma, improved perfusion, and increased chemotherapy sensitivity	Preclinical	[166]
	HA depletion	PEGPH20 (pegylated hyaluronidase)	Autochthonous pancreatic ductal adenocarcinoma models	Gemcitabine	Reduced interstitial pressure, re-expanded tumor microvasculature, and enhanced gemcitabine efficacy	Preclinical	[167]
	HA depletion	PEGPH20	Murine pancreatic cancer models	Gemcitabine	Relieved vascular compression, improved drug delivery, and suppressed tumor growth and prolonged survival	Preclinical	[168]
	HA depletion	PEGPH20	Metastatic pancreatic ductal adenocarcinoma; patients	Gemcitabine	Showed preliminary antitumor activity, particularly in HA-high tumors	Phase Ib; thromboembolic toxicity	[169]
	HA depletion	PEGPH20	Metastatic pancreatic ductal adenocarcinoma; patients	Nab-paclitaxel plus gemcitabine	Improved PFS in the HA-high subgroup	Phase II; thromboembolic events and muscle spasms	[170]
	HA depletion	PEGPH20	Metastatic pancreatic ductal adenocarcinoma; patients	Nab-paclitaxel plus gemcitabine	Failed to improve PFS or OS in biomarker-selected patients	Phase III; increased fatigue, muscle spasms, and hyponatremia	[171]
CAF activation pathway inhibition	TGF-β inhibition	TGF-β blockade	Murine EMT6 breast cancer model	Anti-PD-L1	Fibroblast TGF-β signaling was associated with T-cell exclusion; TGF-β inhibition combined with anti-PD-L1 therapy promoted T-cell infiltration and tumor regression.	Preclinical	[172]
	TGF-β inhibition	TGF-β blockade	Metastatic colorectal cancer model	Anti-PD-1/PD-L1	Restored cytotoxic T-cell responses, prevented metastasis, and sensitized liver metastases to anti-PD-1/PD-L1 therapy	Preclinical	[173]
	TGF-β neutralization	TGF-β-neutralizing antibody	Murine breast and colorectal cancer models	Anti-PD-1	Reduced the frequency and activity of a CAF subset, induced an interferon-responsive fibroblast population, and enhanced antitumor immunity and anti-PD-1 efficacy	Preclinical	[179]
	TGF-β receptor I inhibition	Galunisertib	Unresectable pancreatic cancer; patients	Gemcitabine	Prolonged overall survival compared with gemcitabine alone	Phase II; limited additional toxicity	[180]
	Hedgehog pathway inhibition	IPI-926	Murine pancreatic ductal adenocarcinoma models	Gemcitabine	Transiently depleted desmoplastic stroma, increased tumor vascularity and gemcitabine delivery, and produced transient disease stabilization	Preclinical	[181]
	Hedgehog pathway inhibition	Shh ablation or SMO inhibition	Murine pancreatic ductal adenocarcinoma models	Monotherapy	Reduced stromal abundance but promoted poorly differentiated, highly vascularized, and rapidly progressing tumors	Preclinical; disease-acceleration risk	[182]
	Hedgehog pathway inhibition	Vismodegib	Metastatic pancreatic cancer; patients	Gemcitabine	Failed to improve objective response rate, PFS, or OS	Phase Ib/II; no unexpected toxicity	[183]
	NOX4 inhibition	Setanaxib	Head and neck and colorectal cancer; preclinical models	Monotherapy	Reversed the myCAF phenotype, reduced α-SMA⁺ CAF accumulation, and inhibited tumor growth	Preclinical	[184]
	NOX4 inhibition	Setanaxib	Lung, colorectal, and breast cancer; murine models	HPV E7 vaccine, anti-PD-1, or anti-CTLA-4 therapy	Increased intratumoral CD8⁺ T-cell infiltration and restored immunotherapy responsiveness	Preclinical	[185]
	NOX1/4 inhibition	Setanaxib	Intrahepatic cholangiocarcinoma models	Monotherapy	Impaired CAF function and reduced tumor growth	Preclinical	[186]
	NOX1/4 inhibition	Setanaxib	Recurrent or metastatic HNSCC; patients	Pembrolizumab	Did not improve the primary tumor-size endpoint but was associated with longer PFS and OS and increased intratumoral CD8⁺ T-cell infiltration	Phase II; no new safety signals	[187]

### Conventional CAF-targeted approaches: opportunities and limitations

#### CAF depletion and suppression strategies

Early CAF-targeting strategies primarily focused on eliminating or functionally suppressing tumor-promoting CAF populations, with FAP emerging as the most extensively investigated therapeutic target due to its enrichment in activated CAF subsets across solid tumors. Diverse FAP-directed approaches, ranging from antibody-based targeting and enzymatic inhibition to vaccination, photoimmunotherapy, and engineered immune cell therapies, have since been developed to modulate the CAF compartment.

Monoclonal antibodies have been explored clinically for FAP targeting. Scott et al. conducted a phase I trial of sibrotuzumab (F19), a humanized monoclonal antibody against FAP, in patients with advanced solid tumors. Although sibrotuzumab demonstrated effective localization to FAP-expressing tumor stroma and acceptable safety, it showed limited antitumor activity, suggesting that antibody-mediated FAP targeting alone was insufficient to achieve durable therapeutic benefit [149]. Subsequently, talabostat (Val-boroPro), an inhibitor of FAP-associated enzymatic activity, was evaluated as a pharmacological approach to suppress FAP function. In a phase II trial of patients with metastatic colorectal cancer, talabostat reduced circulating FAP activity but resulted in limited disease stabilization and no objective responses. These findings indicated that suppressing FAP-associated enzymatic activity alone may not be sufficient to overcome CAF-mediated tumor support [150].

Given the limited efficacy of antibody-based targeting and enzymatic inhibition, FAP-based vaccination emerged as an immune-mediated strategy for targeting FAP⁺ CAFs. Loeffler et al. provided early proof of concept using an oral FAP DNA vaccine, which induced CD8⁺ T-cell-mediated depletion of FAP⁺ fibroblasts, reduced collagen deposition, and improved intratumoral drug uptake in murine colon and breast cancer models [151]. Building on this approach, Duperret et al. developed a synthetic consensus FAP DNA vaccine with an enhanced capacity to overcome immune tolerance, inducing both CD8⁺ and CD4⁺ T-cell responses and synergizing with tumor antigen-specific vaccination. However, the efficacy of FAP vaccination remained constrained by the immunosuppressive TME [152]. To address this limitation, Xia et al. combined a FAP-targeted prime-boost vaccine with cyclophosphamide, which strengthened cytotoxic T-cell responses, reduced immunosuppressive stromal components, and improved tumor control in a breast cancer model [153].

In conclusion, these studies established the feasibility of vaccine-mediated targeting of FAP⁺ CAFs. However, therapeutic efficacy remained constrained by immune tolerance and insufficient endogenous antitumor responses. In this context, engineered immune cell therapies have been developed to achieve more sustained and controllable targeting of FAP, most notably through FAP-specific chimeric antigen receptor (CAR) T-cell therapy. By genetically modifying T cells to express a FAP-specific CAR, this strategy enables direct recognition and elimination of FAP⁺ CAFs. In murine tumor models, Tran et al. showed that FAP-specific CAR-T cells effectively depleted CAFs, disrupted the tumor stroma, and promoted CD8⁺ T-cell infiltration, but also induced severe bone toxicity because of FAP expression in normal mesenchymal cells [154]. In another study, Wang et al. developed murine FAP-specific CAR-T cells containing 4-1BB and CD3ζ signaling domains, which inhibited tumor growth and enhanced endogenous CD8⁺ T-cell responses with minimal off-tumor toxicity [155]. This strategy was further extended through sequential combination with CLDN18.2-targeted CAR T-cell therapy, in which prior depletion of FAP⁺ CAFs suppressed MDSC recruitment, promoted the intratumoral persistence of CD8⁺ T cells and CAR T cells, and enhanced antitumor activity in pancreatic cancer models [156]. To further improve the safety and applicability of this approach, a human/murine cross-reactive FAP-specific CAR T-cell construct incorporating 4-1BB costimulatory signaling was developed, showing potent activity against FAP⁺ tumor cells and CAFs with low-level on-target/off-tumor toxicity across multiple preclinical models [157]. Clinical translation was evaluated in the phase I FAPME trial (NCT01722149), in which three patients with malignant pleural mesothelioma received intrapleural FAP-targeted CAR T cells. Two thromboembolic events occurred but were considered unrelated to the treatment, and no treatment-related serious toxicity was reported. Although the trial supported the feasibility and preliminary safety of this approach, its antitumor efficacy could not be determined because of the small sample size [158].

Overall, these studies indicate that the clinical benefit of FAP-directed therapies remains limited despite promising antitumor effects in preclinical models. Given the marked functional heterogeneity of CAFs, broad depletion may also remove fibroblast subsets with tumor-restraining or tissue-protective roles. These limitations have prompted growing interest in targeting specific stromal products and tumor-supportive functions, including collagen deposition and remodeling within the ECM, rather than eliminating the CAF compartment as a whole [159].

#### ECM remodeling strategies

The ECM is a dynamic, cell-derived network of proteins and polysaccharides that provides structural support while transmitting biochemical and mechanical cues within tissues. In the TME, CAFs are major drivers of ECM production and remodeling, and dysregulation of these processes increases matrix density and stiffness, compresses tumor vessels, and limits both therapeutic penetration and immune cell infiltration. These changes promote tumor progression, immune exclusion, and treatment resistance [160]. Accordingly, ECM-directed strategies have focused on reducing collagen deposition and cross-linking and depleting excess hyaluronic acid (HA), with the goal of restoring stromal homeostasis rather than indiscriminately depleting CAFs.

Among ECM-directed approaches, strategies targeting collagen have focused on either limiting its accumulation or preventing its enzymatic cross-linking. Losartan, an angiotensin II type 1 receptor blocker with antifibrotic activity, reduced collagen I production by CAFs and stromal collagen accumulation, improving the tumor penetration and antitumor activity of oncolytic herpes simplex virus and liposomal doxorubicin [161]. Subsequent work showed that losartan also reduced stromal collagen and hyaluronan production, relieved tumor vessel compression, and improved vascular perfusion as well as drug and oxygen delivery, thereby enhancing chemotherapy efficacy and reducing tumor hypoxia [162].

Lysyl oxidase (LOX) family enzymes have emerged as therapeutic targets because of their central role in covalent collagen cross-linking and matrix stiffening. In preclinical models, reducing LOX-dependent cross-linking attenuated ECM stiffening, focal adhesion formation, and PI3K signaling, suppressing mammary tumor progression [163]. Similarly, the LOXL2-specific antibody AB0023 reduced activated fibroblasts, desmoplasia, and tumor-associated vascular remodeling, while inhibiting primary and metastatic tumor growth in xenograft models [164].

Despite these encouraging preclinical results, simtuzumab failed to improve progression-free survival (PFS), overall survival (OS), or the objective response rate when combined with first-line gemcitabine in a randomized phase II trial in patients with metastatic pancreatic adenocarcinoma [165]. More recently, pan-LOX inhibition has emerged as an alternative strategy. In preclinical pancreatic cancer models, the orally bioavailable inhibitor PXS-5505 softened the fibrotic stroma, improved tumor perfusion, and increased sensitivity to chemotherapy. Antifibrotic activity was also observed in patient-derived xenograft (PDX) models [166]. These studies support LOX-dependent collagen cross-linking as a tractable stromal target while highlighting the limited clinical efficacy of selective LOXL2 inhibition. Whether broader LOX blockade can achieve durable stromal remodeling and meaningful clinical benefit remains uncertain.

HA is a major glycosaminoglycan component of the ECM. Its excessive accumulation increases interstitial pressure, compresses tumor vessels, and impairs drug penetration. In autochthonous PDAC models, enzymatic depletion of stromal HA alleviated desmoplastic compression, normalized interstitial fluid pressure, re-expanded the microvasculature, and enhanced the antitumor efficacy of gemcitabine [167]. Similarly, PEGPH20-mediated HA depletion relieved vascular compression and improved drug delivery, while also increasing endothelial fenestration and interendothelial junctional gaps. When combined with gemcitabine, PEGPH20 suppressed tumor growth and prolonged survival in pancreatic cancer models [168]. Encouraged by these preclinical findings, PEGPH20 was evaluated clinically in metastatic PDAC. A phase Ib study combining PEGPH20 with gemcitabine showed preliminary activity, particularly in patients with HA-high tumors, although thromboembolic toxicity emerged as an important concern [169]. This biomarker-associated signal was subsequently explored in the randomized phase II HALO-202 trial, in which the addition of PEGPH20 to nab-paclitaxel and gemcitabine produced a greater PFS benefit in the HA-high subgroup [170]. However, the subsequent phase III HALO-301 trial failed to improve PFS or OS in biomarker-selected patients, ultimately limiting the clinical development of this strategy [171]. Overall, these studies illustrate both the therapeutic potential and the limitations of directly modifying the tumor matrix. Although collagen- and HA-targeted approaches can alleviate stromal compression and improve drug delivery in preclinical models, their clinical benefits have remained inconsistent, underscoring the need to target the signaling programs that sustain pathological CAF activation.

#### CAF activation pathway inhibition

Given the central role of CAF activation in driving tumor progression and treatment resistance, blocking the signaling pathways that sustain this state represents a promising therapeutic strategy. Among these pathways, TGF-β signaling has emerged as a major therapeutic target owing to its pivotal role in fibroblast activation. Evidence from metastatic urothelial cancer and preclinical breast and colorectal cancer models linked fibroblast-associated TGF-β signaling to an immune-excluded phenotype characterized by restricted T-cell infiltration and impaired effector function. TGF-β inhibition promoted T-cell infiltration and enhanced responsiveness to anti-PD-L1 therapy, resulting in tumor regression [172, 173]. These findings have further motivated the development of bispecific and bifunctional antibodies that simultaneously target TGF-β and immune checkpoint signaling [174, 175]. Preclinical studies showed that dual targeting of TGF-β and PD-L1 enhanced T-cell infiltration and antitumor activity, while simultaneous TGF-β/VEGF blockade also potentiated the response to PD-1 inhibition [176–178].

Neutralization of TGF-β also altered the composition and functional state of the fibroblast compartment. In murine carcinoma models, this intervention markedly reduced the abundance and activity of myCAFs while promoting the emergence of a distinct fibroblast population characterized by strong interferon responsiveness and enhanced immunomodulatory properties. These changes were associated with more effective antitumor immunity and increased responsiveness to anti-PD-1 therapy [179]. Galunisertib is an orally available small-molecule inhibitor of the TGF-β receptor type I kinase. In a randomized phase II trial in patients with unresectable pancreatic cancer, galunisertib combined with gemcitabine prolonged overall survival compared with gemcitabine alone, with limited additional toxicity [180].

Beyond TGF-β, Hedgehog signaling is another key regulator of CAF activation. Pharmacological inhibition of Hedgehog signaling with the Smoothened (SMO) inhibitor IPI-926 transiently depleted the desmoplastic stroma, increased tumor vascularity, and enhanced gemcitabine delivery, resulting in transient disease stabilization in PDAC [181]. However, Hedgehog-dependent stroma may also restrain tumor progression. Genetic ablation of tumor-derived sonic hedgehog (Shh) or pharmacological inhibition of SMO reduced stromal abundance but resulted in poorly differentiated, highly vascularized, and rapidly proliferating tumors, suggesting that excessive stromal depletion may accelerate PDAC progression [182]. In a randomized phase Ib/II trial in patients with metastatic pancreatic cancer, vismodegib plus gemcitabine failed to improve objective response rate, PFS, or OS compared with placebo plus gemcitabine [183].

In addition to these canonical pathways, redox signaling also contributes to sustained CAF activation. NADPH oxidase 4 (NOX4), a downstream effector of TGF-β signaling, is a key regulator of the myCAF phenotype. Genetic or pharmacological inhibition of NOX4 attenuated CAF activation and tumor growth across multiple cancer types, supporting its potential as a broadly applicable stromal target [184]. Subsequent work further showed that NOX4 inhibition could revert established CAFs toward a more quiescent phenotype. The NOX1/4 inhibitor setanaxib increased intratumoral CD8⁺ T-cell infiltration, counteracted CAF-mediated immune exclusion, and restored responsiveness to immunotherapy in CAF-rich tumors (Fig. 4B) [185]. More recently, dual NOX1/NOX4 inhibition with setanaxib was shown to impair TGF-β-induced fibroblast transdifferentiation and suppress CAF functions, resulting in reduced tumor growth in vitro and in experimental models of intrahepatic cholangiocarcinoma [186]. Setanaxib was subsequently evaluated in combination with pembrolizumab in a randomized, double-blind phase II trial in patients with recurrent or metastatic HNSCC whose tumors contained CAF levels of at least 5%. Although the combination did not improve the primary tumor-size endpoint, it was associated with longer PFS and OS and increased intratumoral CD8⁺ T-cell infiltration compared with placebo plus pembrolizumab [187]. Overall, these findings highlight the therapeutic potential of targeting pathways that sustain CAF activation, while also underscoring the challenges posed by CAF heterogeneity, context-dependent stromal functions, and limited clinical translation.

### Targeting CAF metabolic reprogramming: emerging opportunities and challenges

Compared with strategies that directly deplete CAFs or broadly disrupt the stromal compartment, metabolic targeting offers a function-oriented approach to suppressing their tumor-supportive and immunosuppressive activities. This strategy may preserve fibroblast populations with tissue-protective or tumor-restraining functions. Targeting key metabolic enzymes, transporters, or metabolite exchange may disrupt tumor-supportive metabolic crosstalk, reduce the accumulation of immunosuppressive metabolites, and restore immune cell function [188, 189]. These effects provide a strong rationale for combining metabolic interventions with immunotherapy. Representative strategies targeting CAF metabolism are summarized in Table 3.

**Table 3:** Therapeutic strategies targeting CAF metabolic reprogramming

Therapeutic category	Target/pathway	Intervention	Evidence	ICB combination	Key findings	Stage/Safety	Ref.
Metabolic barrier disruption	GLUT1-dependent glycolysis-CXCL16 axis	BAY-876 (GLUT1 inhibitor)	Soft-tissue sarcoma; in vitro and murine models	No	Reduced CAF glycolysis and CXCL16 secretion, restored CD8⁺ T-cell infiltration, and enhanced chemotherapy sensitivity	Preclinical; human safety unknown	[96]
Metabolic barrier disruption	LINC01711-LDHA-MCT4 axis	SC01711 (LINC01711 siRNA); VB124 (MCT4 inhibitor)	Lung adenocarcinoma; patient samples, in vitro and murine models	anti-PD-1	Reduced lactate export; restored CD8⁺ T-cell infiltration and function; enhanced anti-PD-1 efficacy	Preclinical; human safety unknown	[97]
Metabolic barrier disruption	CAF-CD73-dependent adenosine production-A2A/A2B receptor signaling	CD73-neutralizing antibody plus A2A/A2B receptor antagonists	Colorectal cancer; patient samples and murine CAF-rich tumor models	No	Blocked adenosine production and signaling; disrupted the CAF-CD73 feedforward circuit; enhanced antitumor immunity	Preclinical; human safety unknown	[99]
Nutrient and metabolite reprogramming	Arginase-mediated L-arginine depletion	BEC hydrochloride (arginase inhibitor)	Melanoma; human melanoma-associated fibroblast/CTL coculture	No	Prevented arginase-dependent TIGIT and BTLA upregulation but did not fully restore CD69, granzyme B, or CTL killing	In vitro; human safety unknown	[100]
Metabolic-epigenetic regulation	NNMT-EZH2-ANGPTL4 axis	Fibroblast-specific NNMT knockout and NNMT-targeted silencing	Hepatocellular carcinoma; patient samples, organoids, and murine models	anti-PD-L1	Restored cytotoxic CD8⁺ T-cell activity and enhanced anti-PD-L1 efficacy	Preclinical; human safety unknown	[102]
Metabolic-epigenetic regulation	CAF NNMT-SAA-TAM recruitment axis	5-Amino-1-methylquinolinium iodide (NNMT inhibitor)	Urothelial bladder cancer; patient samples, in vitro assays, and murine models	anti-PD-L1	Reduced macrophage infiltration and tumor growth; enhanced anti-PD-L1 efficacy	Preclinical; human safety unknown	[121]
Metabolic-epigenetic regulation	NNMT-H3K27me3-complement-MDSC recruitment axis	NNMTi (NCGC00685960) or fibroblast-specific NNMT knockout	Ovarian cancer; patient spatial/single-cell data and multiple murine tumor models	anti-PD-1 and anti-CD47	Reduced complement secretion and MDSC infiltration; enhanced CD8⁺ T-cell activity and restored ICB efficacy	Preclinical; human safety unknown	[22]
Nutrient and metabolite reprogramming	HMGCS2-dependent ketogenesis-KLF5-CXCL12 axis	Ketogenic diet, HMGCS2 overexpression, or β-hydroxybutyrate supplementation	Colorectal cancer; in vitro assays and murine models	anti-PD-1	Restored CAF ketogenesis; reduced CXCL12 production; increased NK- and cytotoxic T-cell infiltration; enhanced anti-PD-1 efficacy	Preclinical; human safety unknown	[104]
Iron metabolism and ferroptosis regulation	CAF-derived FSTL1-DIP2A/p38-NCOA4 ferritinophagy	Deferoxamine plus an FSTL1-neutralizing antibody	Gastric cancer; patient samples, in vitro assays, and patient-derived organoids	No	Reduced NK-cell iron accumulation and ferroptosis; restored NK-cell cytotoxicity	Preclinical; combination safety in patients unknown	[107]
Iron metabolism and ferroptosis regulation	HMOX1-mediated heme degradation-iron-KDM6B axis	Zinc protoporphyrin, deferasirox, GSK-J4, or KDM6B silencing	Prostate cancer; patient samples, in vitro CAF assays, and murine models	No	Reduced myeloid-recruiting factors and suppressive myeloid-cell infiltration; restored CD8⁺ T cells; inhibited tumor growth	Preclinical; human safety unknown	[124]
Iron metabolism and ferroptosis regulation	ZEB1-SLC7A11/system Xc⁻ axis	CAF-specific SLC7A11 knockdown	Prostate cancer; in vitro CAF assays and murine models	No	Increased CAF susceptibility to ferroptosis; reduced immunosuppression and tumor growth	Preclinical genetic intervention; human safety unknown	[125]

Current evidence does not demonstrate that metabolic targeting is broadly superior to conventional strategies based on CAF depletion, ECM remodeling, or inhibition of fibroblast-activating pathways. Most metabolic interventions remain at the preclinical stage, and their efficacy is likely to depend on tumor type, CAF composition, spatial context, and the dominant metabolic dependencies of individual tumors. Moreover, the same metabolic pathway may exert distinct or even opposing functions across CAF subsets and disease stages. Metabolic targeting should therefore be viewed as a context-dependent strategy for functional CAF reprogramming rather than a broadly applicable replacement for existing stromal interventions [190].

A major obstacle to clinical translation is the limited cellular specificity of most metabolic targets. Many metabolic enzymes, membrane transporters, and metabolite receptors involved in CAF function are also widely expressed in malignant cells, immune cells, and normal tissues. Systemic inhibition may therefore disrupt metabolic homeostasis or inadvertently suppress antitumor immunity [191]. Unlike direct depletion strategies such as FAP-targeted CAR T cells, which may damage normal stromal populations [154], the toxicities of metabolic interventions are likely to be broader and less cell-restricted, necessitating careful pharmacodynamic assessment and long-term safety monitoring.

The metabolic plasticity of CAFs may further limit the durability of single-target interventions. When one pathway is inhibited, CAFs may maintain their tumor-supportive functions by switching substrates, activating compensatory metabolic routes, or re-establishing metabolic exchange with neighboring cells. Such adaptation may drive primary or acquired resistance and limit the efficacy of monotherapy. More feasible strategies may therefore include co-targeting complementary metabolic pathways, combining metabolic inhibitors with immunotherapy or stromal remodeling, and developing CAF-selective delivery systems [192].

Overall, metabolic targeting offers a function-oriented alternative to conventional CAF-depletion approaches, with the potential to disrupt tumor-promoting CAF activities while preserving beneficial fibroblast populations. However, its clinical advantages remain unproven. Future studies should prioritize the identification of CAF-specific metabolic targets that can be inhibited without compromising normal tissues or antitumor immunity. Patient selection should also incorporate CAF subtypes, metabolic biomarkers, and immune phenotypes. In parallel, pharmacokinetics, systemic toxicity, immune effects, and compensatory metabolic responses should be evaluated in models that more closely recapitulate human physiology. These efforts will be essential to determine whether CAF-directed metabolic interventions can achieve safe, durable, and predictable clinical benefit.

## Conclusions and future perspectives

Metabolic reprogramming of CAFs is increasingly recognized as a central link between stromal remodeling, immune regulation, and therapeutic resistance. By altering nutrient uptake and utilization, reshaping metabolite production and exchange, and regulating ECM synthesis and organization, CAFs impose sustained structural and metabolic constraints on the TME. These changes restrict the infiltration and function of effector immune cells while supporting immunosuppressive myeloid populations, regulatory lymphocytes, and other tumor-promoting cellular states. CAF metabolic reprogramming should therefore be viewed not as an isolated intracellular event, but as an integral determinant of the tumor immune ecosystem and therapeutic response.

Despite growing interest in this field, the causal relationships between CAF metabolic states and immune phenotypes remain insufficiently defined. Current CAF classifications are largely based on transcriptional features, whereas the stability of the corresponding metabolic phenotypes, their reproducibility across tumor types, and the reversibility of transitions between distinct states remain unclear. Moreover, the same metabolic program may exert different or even opposing effects depending on disease stage, spatial location, and treatment context [193]. Analyses based on a single marker or time point may therefore fail to capture the dynamic functional states of CAFs.

Future studies should move beyond static associations toward spatially and temporally resolved functional validation [63]. Integrating spatial transcriptomics, spatial metabolomics, single-cell multi-omics, and stable-isotope tracing may reveal nutrient competition and metabolite exchange between CAFs, malignant cells, and immune cells, while defining how these interactions evolve during tumor progression and in response to therapy. Key mechanisms should also be validated using patient-derived organoids, three-dimensional co-culture systems, humanized models, and other platforms that more closely recapitulate human physiology. Coupling these approaches with biomarker frameworks that integrate CAF subtypes, metabolic features, and immune phenotypes will be essential for translating current largely associative observations into clinically actionable therapeutic strategies.

## Data Availability

Not applicable.

## Abbreviations

A2BR: A2B adenosine receptor
apCAFs: Antigen-presenting cancer-associated fibroblasts
CAFs: Cancer-associated fibroblasts
DCs: Dendritic cells
ECM: Extracellular matrix
EMT: Epithelial-to-mesenchymal transition
EndMT: Endothelial-to-mesenchymal transition
EVs: Extracellular vesicles
HCC: Hepatocellular carcinoma
HIF-1α: Hypoxia-inducible factor-1α
HNSCC: Head and neck squamous cell carcinoma
iCAFs: Inflammatory cancer-associated fibroblasts
ICB: Immune checkpoint blockade
ICIs: Immune checkpoint inhibitors
IDO: Indoleamine 2,3-dioxygenase
IFN-γ: Interferon-γ
LAMs: Lipid-associated macrophages
MDSCs: Myeloid-derived suppressor cells
MSCs: Mesenchymal stem cells
myCAFs: Myofibroblastic cancer-associated fibroblasts
NETs: Neutrophil extracellular traps
NK: Natural killer
NNMT: Nicotinamide N-methyltransferase
OS: Overall survival
OSCC: Oral squamous cell carcinoma
PD-1: Programmed cell death protein 1
PD-L1: Programmed death-ligand 1
PDAC: Pancreatic ductal adenocarcinoma
PDX: Patient-derived xenograft
PFS: Progression-free survival
PSCs: Pancreatic stellate cells
scRNA-seq: Single-cell RNA sequencing
TAMs: Tumor-associated macrophages
TANs: Tumor-associated neutrophils
TCA: Tricarboxylic acid
TGF-β: Transforming growth factor-β
TLSs: Tertiary lymphoid structures
TME: Tumor microenvironment
TNBC: Triple-negative breast cancer
Tregs: Regulatory T cells
TRFs: Tissue-resident fibroblasts
